# Characterizing Thrombotic Complication Risk Factors Associated With COVID-19 via Heterogeneous Patient Data: Retrospective Observational Study

**DOI:** 10.2196/35860

**Published:** 2022-10-21

**Authors:** Bedda Rosario, Andrew Zhang, Mehool Patel, Amol Rajmane, Ning Xie, Dilhan Weeraratne, Gil Alterovitz

**Affiliations:** 1 IBM Round Rock, TX United States; 2 Biomedical Cybernetics Laboratory Brigham and Women's Hospital Boston, MA United States; 3 IBM Akron, OH United States; 4 IBM Palo Alto, CA United States; 5 IBM Boston, MA United States

**Keywords:** COVID-19, thrombotic complications, logistic regression, EHR, electronic health record, insurance claims data

## Abstract

**Background:**

COVID-19 has been observed to be associated with venous and arterial thrombosis. The inflammatory disease prolongs hospitalization, and preexisting comorbidities can intensity the thrombotic burden in patients with COVID-19. However, venous thromboembolism, arterial thrombosis, and other vascular complications may go unnoticed in critical care settings. Early risk stratification is paramount in the COVID-19 patient population for proactive monitoring of thrombotic complications.

**Objective:**

The aim of this exploratory research was to characterize thrombotic complication risk factors associated with COVID-19 using information from electronic health record (EHR) and insurance claims databases. The goal is to develop an approach for analysis using real-world data evidence that can be generalized to characterize thrombotic complications and additional conditions in other clinical settings as well, such as pneumonia or acute respiratory distress syndrome in COVID-19 patients or in the intensive care unit.

**Methods:**

We extracted deidentified patient data from the insurance claims database IBM MarketScan, and formulated hypotheses on thrombotic complications in patients with COVID-19 with respect to patient demographic and clinical factors using logistic regression. The hypotheses were then verified with analysis of deidentified patient data from the Research Patient Data Registry (RPDR) Mass General Brigham (MGB) patient EHR database. Data were analyzed according to odds ratios, 95% CIs, and *P* values.

**Results:**

The analysis identified significant predictors (*P*<.001) for thrombotic complications in 184,831 COVID-19 patients out of the millions of records from IBM MarketScan and the MGB RPDR. With respect to age groups, patients 60 years and older had higher odds (4.866 in MarketScan and 6.357 in RPDR) to have thrombotic complications than those under 60 years old. In terms of gender, men were more likely (odds ratio of 1.245 in MarketScan and 1.693 in RPDR) to have thrombotic complications than women. Among the preexisting comorbidities, patients with heart disease, cerebrovascular diseases, hypertension, and personal history of thrombosis all had significantly higher odds of developing a thrombotic complication. Cancer and obesity were also associated with odds>1. The results from RPDR validated the IBM MarketScan findings, as they were largely consistent and afford mutual enrichment.

**Conclusions:**

The analysis approach adopted in this study can work across heterogeneous databases from diverse organizations and thus facilitates collaboration. Searching through millions of patient records, the analysis helped to identify factors influencing a phenotype. Use of thrombotic complications in COVID-19 patients represents only a case study; however, the same design can be used across other disease areas by extracting corresponding disease-specific patient data from available databases.

## Introduction

The World Health Organization reported over 270 million positive cases for COVID-19 and over 5.3 million deaths from the virus worldwide as of December 14, 2021 [1]. As infected patients demonstrate vastly different outcomes, it is critical to identify key patient characteristics that govern the course of the disease across large patient cohorts as early as possible to help allocate the right resources and improve patient outcomes [2]. Logistic regression and machine-learning algorithms have been used to predict which COVID-19 patients will require hospitalization and intensive care to ensure that resources are prioritized to individuals with the highest risk [3-7]. Many of these algorithms make use of routinely collected clinical data.

Although it is well-established that COVID-19 is associated with respiratory complications, the disease has also been observed to cause venous and arterial thrombosis [8]. A hyperinflammatory response has been associated with COVID-19 in increasing the risk of thrombosis [9]. The inflammatory disease process, prolonged hospitalization, and preexisting comorbidities can all contribute to the aggressive thrombotic burden in patients with thrombosis [10-13]. A study in two Dutch university hospitals and one Dutch teaching hospital showed a 31% incidence of thrombotic complications in patients in the intensive care unit (ICU) with COVID-19 [14]. Similarly, the incidence of venous thromboembolism (VTE) in ICU patients was reported to be 25% at Union Hospital, Wuhan, China [15]. In general, VTE has been found to affect up to 46% of hospitalized patients with COVID-19 [16], and a meta-analysis suggested that COVID-19 patients with thrombotic complications have a 2.1-fold higher risk of mortality than those without thrombotic complications [17]. However, VTE and other related vascular complications may go unnoticed in critical care settings [18,19]. As such, early risk stratification is clinically critical for the COVID-19 patient population [20].

There are several potential hypotheses on the mechanisms that may be associated with or responsible for thrombotic complications. For example, there is some preliminary evidence that autoimmune reactions may play a role [21]. In addition, drug interactions are treatment challenges introduced by the therapeutic agents available for COVID-19 [22]. As the population of patients recovering from COVID-19 is steadily growing, a systematic study of the sequelae during the postacute COVID-19 phase is important to collect clinical and scientific evidence to determine the best care for these patients. Furthermore, thromboembolic complications have been reported as a part of postacute COVID-19 syndrome [23-25]. Accordingly, the aim of this study was to use real-world data evidence toward building the foundation for development of a software system that systematically identifies factors affecting VTE in COVID-19 patients.

Electronic health records (EHRs) are widely becoming adopted in health care systems with increasing capability of record sharing across different organizations [22]; however, there remain constraints in using such data along with challenges in gaining unrestricted access. Insurance claims data capture information from all doctors and providers, whereas EHR data capture only the portion of care provided by doctors using the EHR. However, insurance claims data also have limitations such as that these data only cover insured patients. We aimed to bridge these gaps between EHR and claims data, and accommodate both data sources to take advantage of a wider range of data. This design was particularly useful to synthesize a hypothesis regarding COVID-19 and thrombotic complications from IBM and Mass General Brigham (MGB; Boston, Massachusetts) data using IBM’s MarketScan claims data set, which was cross-verified with MGB’s EHR-derived database. This analysis thus provided a useful approach to bridge the gap with EHR data sets without requiring Health Insurance Portability and Accountability Act–level individual patient information, thereby avoiding the multiple-step process for access.

During the global COVID-19 pandemic, collaboration between organizations has accelerated understanding of the SARS-CoV-2 virus and the COVID-19 disease it causes. While EHR data are widely used in COVID-19 retrospective studies [6,22-26], some organizations use proprietary databases. We have been working to design a method that can handle different types of health care data storage, including the standardized EHR databases as well as any other proprietary data sources such as the insurance claims database used in this study. To respect patient privacy concerns, we only used deidentified patient data when querying the databases. These measures were chosen to make it easier for the work to potentially be used for global collaboration in the COVID-19 pandemic and other cases. This research characterizes thrombotic complication risk factors associated with COVID-19 using information from EHR and insurance claims databases. Comprehensive treatment guidelines and reviews can be found in prior literature [27,28].

## Methods

### Data Collection

This retrospective observational study utilized deidentified data from IBM’s MarketScan commercial claims database. These data were compared and validated with data from the MGB EHR. Adult patients with a COVID-19 diagnosis between February 1, 2020, and September 30, 2020, were included in the study. Patient demographics included age, gender, ethnicity (EHR database only), and geographic location. We focused on the following comorbidities: hypertensive disease, diabetes, cancer, respiratory diseases (asthma, acute respiratory distress syndrome, chronic bronchitis, emphysema, bronchiectasis, and chronic obstructive pulmonary disease), heart disease (coronary artery disease, heart failure, cardiomyopathy, atrial fibrillation, and ischemic heart disease), cerebrovascular disease (stroke and cerebrovascular disease), liver disease, kidney disease, prior history of thrombosis, HIV, pregnancy, sleep apnea, tobacco smoking use, and obesity. Interventions included veno-venous extracorporeal membrane oxygenation (ECMO), mechanical ventilation, extraneous oxygen use, and medications. The thrombotic complications focused on ST elevation myocardial infarction (STEMI) and non-STEMI myocardial infarction, pulmonary embolism, cerebral infarction, arterial embolism and thrombosis, other venous embolism and thrombosis, transient ischemic attacks and related syndromes, other acute ischemic heart diseases, and other cerebrovascular diseases.

### Mapping of Diagnosis Codes

This study included patients with a confirmed COVID-19 diagnosis (International Classification of Diseases, Tenth Revision [1CD-10] diagnosis codes U071, B342, Z8616, J1282, B9729) between February 1, 2020, and September 30, 2020. The outcome of interest was a thrombosis diagnosis (ICD-10 diagnosis codes I21, I24, I26, I63, I74, I82, Z8671, M622, and G45) between February 1, 2020, and September 30, 2020.

### Querying Data From the Claims Database

We performed a retrospective analysis of the IBM MarketScan Commercial Database and Medicare Supplemental Database from February 1, 2020, to September 30, 2020, to identify patients. This represents the most recently available data at the time of analysis in IBM MarketScan Treatment Pathways, a cloud-based analytic interface that overlays onto MarketScan Research Databases. MarketScan is one of the largest deidentified longitudinal patient-level health databases in the United States, which includes information on over 39 million individuals, including active employees and their dependents, early retirees, and Consolidated Omnibus Budget Reconciliation Act (COBRA) continuers, insured by approximately 40 employer-sponsored health plans representing all 50 states. A total of 259,470 patients had received a COVID-19 diagnosis at some point between February 1, 2020, and September 30, 2020. Of these, 153,137 patients were continuously enrolled for 2 years prior to the COVID-19 diagnosis and were included in the study.

As an insurance claims database, MarketScan encompasses information from multiple providers in the patient journey with a broader nationwide reach. Insurance claims data provide information on whether a prescription was filled, as opposed to EHR data that only state whether or not a drug was prescribed. MarketScan can effectively complement EHR data by providing an extremely broad view of a patient’s interactions across the continuum of the health care system and by providing access to large and diverse samples.

It should be noted that a few of the individuals may drop in and out of the MarketScan data set due to health insurance coverage changes. Hence, while performing these analyses using MarketScan (or any other claims data set), samples are restricted to patients who are continuously enrolled over the observation period.

### Querying Data From the EHR Database

We gathered patient data from the MGB patient record database Research Patient Data Registry (RPDR), a centralized clinical data registry. The data warehouse includes 6.5 million patients and 2.2 billion rows of clinical data, serving as a central clinical data registry for inpatient and outpatient encounters from various hospital systems to support clinical research.

The RPDR query tool allows for a search for the number of patients at the hospital with a given set of characteristics. We searched for patients at the hospital between February 1, 2020, and September 30, 2020. Patients were characterized using ICD-10 medical codes for the respective medical conditions with a combination of codes to identify COVID-19 patients with thrombosis and potential associated comorbidities. A total of 31,364 patients had received a COVID-19 diagnosis from February 1, 2020, to September 30, 2020, and were included in the study.

### Drawing and Verifying Hypotheses Using Logistic Regression

Descriptive statistics are summarized as frequencies and percentages for categorical data. A simple (or unadjusted) logistic regression model was used to assess the strength of the association between demographic and clinical factors and phenotype. The demographic and clinical factors included demographics, comorbidities, and interventions. In this study, phenotype was defined as a dichotomous variable, and we focused on diagnosis of a thrombotic complication (ie, with or without thrombotic complication).

The results are summarized by the odds ratio (OR), corresponding 95% CI, and *P* value. All tests were 2-sided and the significance level was set to *P*=.001. All statistical analyses were performed using the Modern Applied Statistics with S (MASS) statistical software library version 7.3.54 [29] in R, version 4.1.0 [30].

### Age and Gender Distributions From Patients in the Claims and EHR Data Sets

**Figure 1:**
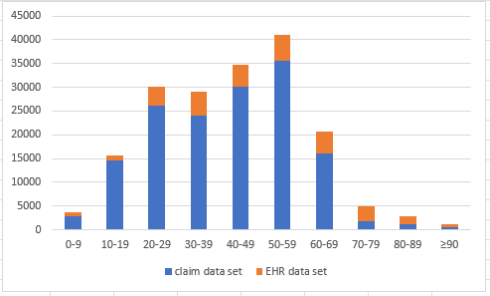
Patients’ age distributions from the insurance claims and electronic health record (EHR) data sets. The x-axis is age and the y-axis is patient count.

Within the study time period, there were 153,137 COVID-19 patients in the claim data, with 44.8% being men. There were 31,364 COVID-19 patients in the EHR data, with 43.9% being men. The age distributions are shown in Figure 1.

### Comorbidity Distributions From Patients in the Claims and EHR Data Sets

**Figure 2:**
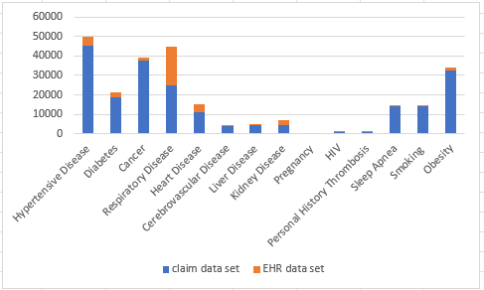
Patients’ comorbidity distributions from the insurance claims and electronic health record (EHR) data sets. The x-axis is comorbidities and the y-axis is patient counts.

COVID-19 patient comorbidity distributions are shown in Figure 2.

### Handling of Missing Data

We encountered two types of missing data. The first involved missing at least one variable in a data set. Given the low rate of such missingness (<2.5% for any individual variable), imputation was deemed unnecessary [28,31]. The other involved missing a category of data in one data set. There were three such cases: ethnicity and lab data are in the EHR data set but not in the claims dataset, whereas region data are in the claims data set but not in the EHR data set (the EHR data set includes patients mostly from the northeast region of the United States). We performed analysis on one data set in the three cases with the understanding that they would not be cross-verified.

### Ethics Approval

This study was approved by the institutional review board of MGB (IRB Protocol #2021P001133).

### Data Analysis

We performed an analysis to determine patients’ clinical and demographic factors associated with thrombotic complications for patients with a COVID-19 diagnosis. Data queried from IBM MarketScan were stored as CSV files. The analysis read from the CSV files and drew hypotheses based on a predefined *P* value threshold (<.001), which was then verified using data queried from the RPDR database.

## Results

### Age and Thrombotic Complications 

To compare the thrombotic complications between the young and old population, we categorized COVID-19 patients into two age groups: those younger than 60 years and those aged 60 years and older. Table 1 lists the frequency (ie, count) of COVID-19 patients with and without thrombotic complications, the calculated *P* value, OR, and the 95% CI from the claims database. The corresponding data from the EHR-compatible database are listed in Table 2. As demonstrated, age and thrombotic complications were significantly associated. In addition, patients aged 60 years and older had a much higher odds to have thrombotic complications. Results from both data sets were consistent despite patients in the two data sets being from different geographical regions and backgrounds. This provided more confidence to the findings and showed how the two data sets could enrich each other.

**Figure 3:**
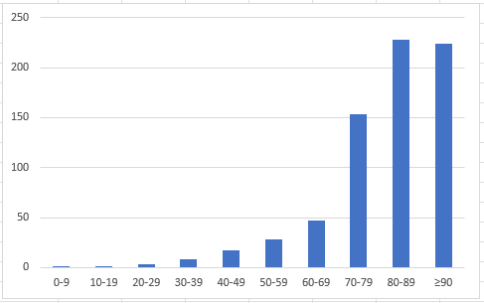
Odds ratio of thrombotic complications with age (*P*<.001). The x-axis is age and the y-axis is the odds ratio.

As shown in Figure 3, with finer age grouping, we also observed that the OR for thrombotic complications consistently increased with age (with the exception that the odds for the age groups of 80-89 years and 90 years and older were similar), with *P*<.001.

**Table 1:** Age and thrombotic complications and the strength of their association based on claims data.

Age group	No thrombotic complication, n	Thrombotic complication, n	Odds ratio (95% CI)	*P* value
<60 years	130,293	3314	Reference	N/A^a^
≥60 years	17,379	2151	4.866 (4.599-5.149)	<.001

^a^N/A: not applicable.

**Table 2:** Age and thrombotic complications and the strength of their association based on electronic health record data.

Age group	No thrombotic complication, n	Thrombotic complication, n	Odds ratio (95% CI)	*P* value
<60 years	20,338	487	Reference	N/A^a^
≥60 years	8796	1339	6.357 (5.714-7.073)	<.001

^a^N/A: not applicable.

### Gender and Thrombotic Complications

Men had higher odds for thrombotic complications when compared to women from both data sets (Tables 3 and 4). Similar to age, the results showed that the two data sets were consistent and enrich each other. This result aligns with prior literature [32].

**Table 3:** Gender and thrombotic complications and the strength of their association based on claims data.

Gender	No thrombotic complication, n	Thrombotic complication, n	Odds ratio (95% CI)	*P* value
Men	65,926	2738	1.245 (1.180-1.314)	<.001
Women	81,188	2727	Reference	N/A^a^

^a^N/A: not applicable.

**Table 4:** Gender and thrombotic complications and the strength of their association based on electronic health record data.

Gender	No thrombotic complication, n	Thrombotic complication, n	Odds ratio (95% CI)	*P* value
Men	12,708	1064	1.693 (1.542-1.859)	<.001
Women	16,763	829	Reference	N/A^a^

^a^N/A: not applicable.

### Comorbidities and Thrombotic Complications

We examined the associations between a thrombotic complication and preexisting conditions such as hypertensive disease, diabetes, cancer, respiratory disease, heart disease, cerebrovascular disease, liver disease, pregnancy, HIV, personal history of thrombosis, sleep apnea, smoking, and obesity. All comorbidities were significantly associated with thrombosis in both data sets (Tables 5 and 6). Although the relative ORs differed in the two data sets, the results were consistent. In both data sets, patients with cerebrovascular disease had the second highest odds to have thrombotic complications, and patients with heart diseases had very similar odds to have a thrombotic complication. In addition, patients with HIV, cancer, and obesity had relatively lower odds to have thrombosis in both data sets. A major difference was that personal history of thrombosis had the highest odds in the claims data set but ranked fifth in the EHR data set. This difference might be due to the small number of patients (n=250) with a personal history of thrombosis in the EHR data set.

**Table 5:** Comorbidity and thrombotic complications and the strength of their association based on claims data.

Comorbidity	No thrombotic complication, n	Thrombotic complication, n	Odds ratio (95% CI)	*P* value
Hypertension	41,513	3890	6.316 (6.950-6.704)	<.001
Diabetes	16,688	1959	4.386 (4.140-4.646)	<.001
Cancer	35,684	2092	1.947 (1.841-2.058)	<.001
Respiratory disease	23,156	1847	2.745 (2.591-2.908)	<.001
Heart disease	8743	2401	12.452 (11.755-13.191)	<.001
Cerebrovascular disease	2542	1406	19.776 (18.399-21.258)	<.001
Liver disease	4044	450	3.187 (2.880-3.527)	<.001
Kidney disease	3316	989	9.619 (8.906-10.389)	<.001
HIV	712	51	1.944 (1.462-2.587)	<.001
History of thrombosis	189	473	73.938 (62.318-87.727)	<.001
Sleep apnea	12,970	1178	2.854 (2.669-3.051)	<.001
Smoking use	13,141	1177	2.810 (2.628-3.005)	<.001
Obesity	30,288	2293	2.802 (2.651-2.961)	<.001

**Table 6:** Comorbidity and thrombotic complications and the strength of their association based on electronic health record data.

Comorbidity	No thrombotic complication, n	Thrombotic complication, n	Odds ratio (95% CI)	*P* value
Hypertension	3079	1172	13.675 (12.373-15.113)	<.001
Diabetes	1894	671	7.853 (7.070-8.723)	<.001
Cancer	890	261	5.048 (4.359-5.845)	<.001
Respiratory disease	17,858	1717	5.891 (5.045-6.879)	<.001
Heart disease	2768	1118	13.661 (12.366-15.093)	<.001
Cerebrovascular disease	294	253	15.053 (12.632-17.937)	<.001
Liver disease	416	160	6.340 (5.25-7.656)	<.001
Kidney disease	2144	718	7.648 (6.902-8.476)	<.001
HIV	60	17	4.368 (2.544-7.499)	<.001
History of thrombosis	159	91	9.154 (7.044-11.896)	<.001
Sleep apnea	395	155	6.454 (5.327-7.820)	<.001
Smoking use	159	223	24.206 (19.633-29.842)	<.001
Obesity	1073	237	3.722 (3.207-4.321)	<.001

### External Intervention and Thrombotic Complications

We examined three external interventions (veno-venous ECMO, mechanical ventilation, and extraneous oxygen use) and their association with thrombotic complication. The ORs and *P* values are summarized in Table 7 and Table 8 for claims and EHR compatible data sets, respectively. Veno-venous ECMO and extraneous oxygen interventions were strongly associated with thrombotic complications in both data sets. Mechanical ventilation was significantly associated with thrombotic complications in the claims data set; however, the number of cases in the EHR compatible data set was too low for appropriate analysis.

**Table 7:** External interventions and thrombotic complications and the strength of their association based on claims data.

External Interventions	No thrombotic complication, n	Thrombotic complication, n	Odds ratio (95% CI)	*P* value
Veno-venous ECMO^a^	34	36	28.794 (18.005-46.047)	<.001
Mechanical ventilation	372	324	24.955 (21.447-29.037)	<.001
Extraneous oxygen use	423	231	15.364 (13.057-18.078)	<.001

^a^ECMO: extracorporeal membrane oxygenation.

**Table 8:** External interventions and thrombotic complications and the strength of their association based on electronic health record data.

External interventions	No thrombotic complication, n	Thrombotic complication, n	Odds ratio (95% CI)	*P* value
Veno-venous ECMO^a^	28	25	13.839 (8.054-23.779)	<.001
Mechanical ventilation	3	3	15.332 (3.092-76.016)	<.001
Extraneous oxygen use	137	56	6.418 (4.687-8.790)	<.001

^a^ECMO: extracorporeal membrane oxygenation.

### Medication Intervention and Thrombotic Complications 

We examined six medication interventions (lopinavir/ritonavir, dexamethasone, remdesivir, monoclonal antibody, tocilizumab, and antimalarials) and their association with thrombotic complication using EHR data. The ORs and *P* values are summarized in Table 9 for the EHR data set. Approximately 1.74% of the COVID-19 patients, the highest proportion in this group, took dexamethasone. A previous report showed that dexamethasone was associated with a reduction in mortality in patients with advanced COVID-19 [33]. Our analysis showed that these patients are 5 times more likely to have thrombotic complications. Approximately 1.26% of the COVID-19 patients, the second highest proportion in this group, took remdesivir. Remdesivir was suggested to be beneficial in shortening the time to recovery in hospitalized COVID-19 patients [34]. Our analysis showed that these patients are also 3 times more likely to have thrombotic complications.

For the claims data set, information was available for three of the above medicines, and the results of this analysis are shown in Table 10. Approximately 2.71% of the COVID-19 patients, the highest proportion in this group, took dexamethasone, and they were 3 times more likely to have thrombotic complications.

**Table 9:** Medication and thrombotic complications and the strength of their association based on electronic health record data.

Medication interventions	No thrombotic complication, n	Thrombotic complication, n	Odds ratio (95% CI)	*P* value
Lopinavir/ritonavir	10	3	4.599 (1.265-16.723)	.02
Dexamethasone	405	134	5.375 (4.396-6.573)	<.001
Remdesivir	325	66	3.185 (2.434-4.168)	<.001
Monoclonal antibody	18	15	12.852 (6.467-25.541)	<.001
Tocilizumab	119	37	4.835 (3.333-7.013)	<.001
Antimalarials	3	3	15.332 (3.093-76.016)	.001

**Table 10:** Medication and thrombotic complications and the strength of their association based on claims data.

Medication interventions	No Thrombotic complication, n	Thrombotic complication, n	Odds ratio (95% CI)	*P* value
Lopinavir/ritonavir	5	3	16.221 (3.876-67.893)	<.001
Dexamethasone	3706	442	3.418 (2.083-3.788)	<.001
Antimalarials	2346	177	2.074 (1.775-2.422)	<.001

### Lab Results and Thrombotic Complications

We examined six lab results that were recorded as abnormal from the EHR data set. The results of the analysis are summarized in Table 11. The claims data set does not have corresponding lab information.

**Table 11:** Strength of associations between lab results and thrombotic complications based on electronic health record data.

Lab result	No thrombotic complication, n	Thrombotic complication, n	Odds ratio (95% CI)	*P* value
D-dimer level	5354	1418	13.174 (11.824-14.677)	<.001
Platelet count	14,279	1807	21.634 (17.404-26.891)	<.001
Prothrombin time	5635	1528	17.344 (15.416-19.513)	<.001
Fibrin degradation products	5544	1208	7.455 (6.758-8.224)	<.001
Fibrinogen	4183	1117	8.533 (7.742-9.405)	<.001
C-reactive protein	12,439	1688	10.95 (9.455-12.682)	<.001

### Ethnicity and Thrombotic Complications

We examined ethnicities and their associations with thrombotic complications using the EHR data set (Table 12). The claims data set does not have ethnicity-related information.

**Table 12:** Strength of associations between ethnicity and thrombotic complications based on electronic health record data.

Ethnicities	No thrombotic complication, n	Thrombotic complication, n	Odds ratio (95% CI)	*P* value
Asian	1007	53	0.800 (0.575-1.012)	.06
Black	3293	285	1.357 (1.181-1.558)	<.001
Hispanic	1770	76	0.606 (0.478-0.768)	<.001
White	17,527	1193	Reference	N/A^a^

^a^N/A: not applicable.

### Region and Thrombotic Complications

The insurance claims data set includes patients from all regions. With *P*<.001, the Northcentral region had the highest OR and the West had the lowest OR for thrombotic complications (Table 13). The EHR data set includes mostly patients in the Northeast where the MGB is located, and therefore the corresponding analysis was not available.

**Figure 4:**
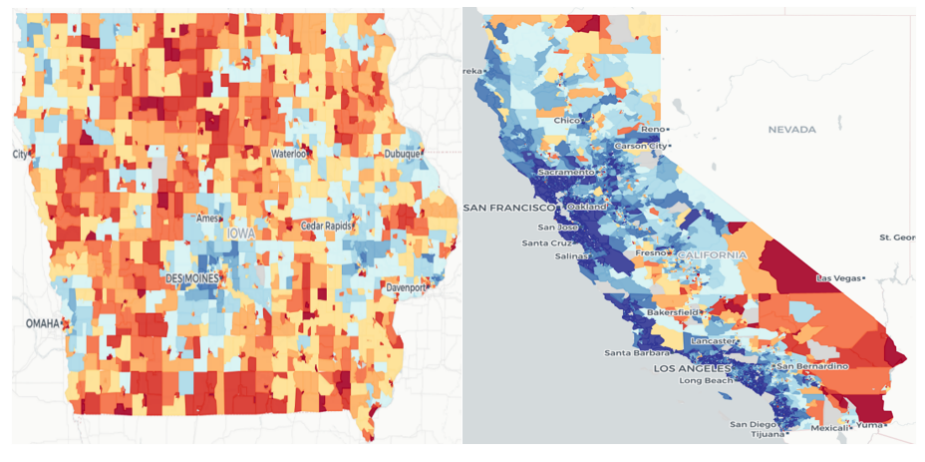
Area Deprivation Index of Iowa (left) and California (right). Deep red indicates the most disadvantaged area and deep blue indicates the least disadvantaged area. Iowa belongs to the Northcentral region where COVID-19 patients have an odds ratio of 1.562 of having thrombotic complications. California belongs to the West region, where COVID-19 patients have an odds ratio of 0.701 of having thrombotic complications.

The regions are divided as shown in Multimedia Appendix 1. Our analysis demonstrated that COVID-19 patients in the Northcentral region of the United States have an OR of 1.562, while patients in the West have an OR of 0.701 to have thrombotic complications. This finding correlates well with the Area Deprivation Index (ADI) of regions [35]. We have included the ADI values of Iowa (Northcentral) and California (West) in Figure 4 to highlight this point.

**Table 13:** Strength of associations between region and thrombotic complications based on insurance claims data.

Region	No thrombotic complication, n	Thrombotic complication, n	Odds ratio (95% CI)	*P* value
Northeast	27,742	1007	Reference	N/A^a^
Northcentral	25,912	1631	1.562 (1.439-1.695)	<.001
South	78,557	2588	0.908 (0.843-0.977)	<.001
West	14,964	381	0.701 (0.622-0.791)	<.001

^a^N/A: not applicable.

## Discussion

### Principal Findings

We found factors related to the demographics, comorbidities, therapeutic interventions, and labs of COVID-19 patients that are strongly associated with the risk of experiencing thrombotic complications. The analysis approach adopted in this study can be leveraged to work across heterogeneous patient databases from different health care and research organizations by using deidentified patient count data. This study used claims and EHR data sets as a case study, but the approach can also be generalized to handle multiple data sources.

The counts were queried with ICD-10 diagnosis codes of the phenotypes being studied. This facilitates collaboration in tackling difficult local and global health issues. In this case study, we analyzed thrombotic complications associated with demographic and clinical factors in COVID-19 patients using insurance claims and EHR databases. We found the design to be very productive in our collaboration where we used claims data to draw hypotheses and EHR data for validation. The two data sets are mostly consistent and enrich each other, except in cases for a very small sample size in EHR-derived data.

The claims and EHR databases have different storage formats, query syntaxes, and security concerns. Our design was to use the common ICD-10 code to run queries on each database and store the query results in CSV files, so that we could use the same R code to read the CSV files and perform the statistical analysis. This also minimized data exchanges between the two geographically dispersed teams.

When selecting factors that are associated with thrombotic complications, we focused on four main categories: demographics, comorbidities, interventions, and lab results. A problem we encountered was that some categories of data might be missing from one data set; for example, the claims database does not have information on all the same prescription drugs as found in the EHR data set. We performed the analysis using only one data set when we deemed the factors of interest to be potentially important. Our analysis of the EHR data set showed that the three most frequently used medications are dexamethasone, remdesivir, and tocilizumab. These medications were associated with thrombotic complications with ORs of 5.375, 3.185, and 4.835, respectively. These were also the medications considered in a previous model developed to predict the requirement of ICU and VTE for COVID-19 patients [28]. Patient lab data, including the D-dimer level, platelet count, prothrombin time, fibrin degradation products, and fibrinogen, are available only in the EHR data set. We believed that these factors are clinically associated with thrombotic complications, which was supported by our analysis results. D-dimer level, one of the top three factors in the lab results category, had an OR of 13 for thrombotic complications, and was also used to predict VTE development in COVID-19 patients in the previous study [28]. All of the top three findings, D-dimer level, platelet count, and prothrombin time, were also previously used in a machine-learning model to predict the need for invasive mechanical ventilation and the mortality of COVID-19 patients [36]. This further validated the strength of the model when applied to large and diverse data sets.

All patients with the preexisting conditions listed in Tables 5 and 6 had much higher odds of having thrombotic complications than other patients in both data sets. It is interesting to note that for patients with underlying cerebrovascular disease, the odds of thrombotic complications in insurance claims data were 19-fold higher and the odds in EHR-derived data were 15-fold higher, ranking second in the comorbidities for COVID-19 patients. It is also interesting to note that for patients with heart disease, the odds for thrombotic complications in both data sets were approximately 13-fold higher, and both ranked third in the comorbidity listings. This further highlights the consistency of the two data sets.

For COVID-19 patients that received external interventions of veno-venous ECMO and extraneous oxygen use, each intervention had much higher odds for thrombotic complications in both data sets.

MarketScan claims data can potentially be very useful in understanding the impact of COVID-19 by monitoring these cases longitudinally to document short-term and long-term patient outcomes.

### Comparison to Prior Work

We found that COVID-19 patients aged 60 years and older were approximately 5 times more likely to have thrombotic complications than those under 60 years old. Although it is well documented that older patients are more susceptible to thrombotic complications [37], this research provides a quantitative measurement of the degree to which this is true in COVID-19 patients.

In terms of gender, men were 1.25 times more likely in the claims data (and 1.69 times more likely in EHR-derived data) to have thrombotic complications compared to women. Although the ORs were slightly different between the two data sets, both showed that men are statistically more likely than women to have thrombotic complications. This finding is consistent with previous studies indicating that men are more likely to be afflicted with thrombotic complications [32].

### Strengths and Limitations

This study used two distinct data sets with 184,831 COVID-19 patients and very comprehensive demographic and clinical information. This allowed us to investigate thrombotic complications from different aspects. We designed an approach that worked with both data sets and found factors strongly associated with thrombotic complications. This approach facilitated teams with different data formats to collaborate. Furthermore, our findings are consistent with the existing literature.

This study focused on patients who received a COVID-19 diagnosis between February 1, 2020, and September 30, 2020, in the United States, and the EHR-derived data included mostly patients in the northeast region of the country. Thus, this data source does not cover the full domestic United States or global perspective. Although we used data from over 184,000 COVID-19 patients and a very small *P* value threshold (*P*<.001) to draw and verify hypotheses on whether a clinical factor affected thrombotic complications, the overall patient count used is relatively small compared with the global patient counts.

We examined factors individually, but it is possible that some factors might be correlated. This was the first phase of the research, and the main goal was to verify the consistency of the two data sets, demonstrating that all factors are associated with thrombotic complications. The second phase of this research will focus on multivariables analysis, as described below. Moreover, this study did not investigate the temporal relationship between interventions and the thrombosis complications. 

### Future Directions

To determine how each factor contributes to a patient’s thrombotic complications, we will explore explainable machine-learning models [38-40] to train models with all the factors we identified in this study. The databases can provide deidentified individual patient data, which can be used to train explainable machine-learning models. The models will not only predict a COVID-19 patient’s risk of thrombotic complications but also determine each factor’s contribution.

### Conclusions

In this work, we examined heterogeneous patient databases and performed an analysis that does not depend on individual patient–level data. This proved to be a valuable approach for collaboration between health care and research organizations with data from different sources, in different storage formats, and with different patient privacy constraints. Via analysis across research collaborators with heterogeneous data sources, we found important demographic and clinical factors associated with thrombotic complications in patients with COVID-19. Our research provides for a collaborative and early risk stratification approach, as a critical step toward helping to ensure efficient resource allocation and better outcomes for the COVID-19 patient population.

## Appendix

Multimedia Appendix 1 Division of US regions.

## Abbreviations

ADI: Area Deprivation Index
COBRA: Consolidated Omnibus Budget Reconciliation Act
ECMO: extracorporeal membrane oxygenation
EHR: electronic health record
ICD-10: International Classification of Diseases, Tenth Revision
ICU: intensive care unit
MASS: Modern Applied Statistics with S
MGB: Mass General Brigham
OR: odds ratio
RPDR: Research Patient Data Registry
STEMI: ST elevation myocardial infarction
VTE: venous thromboembolism
