# Supplementary material for: Characterizing Thrombotic Complication Risk Factors Associated With COVID-19 via Heterogeneous Patient Data: Retrospective Observational Study
Source: J Med Internet Res. 2022 Oct 21;24(10):e35860. doi: 10.2196/35860 (PMC9591707; doi:10.2196/35860)
Supplement: Multimedia Appendix 1 [file jmir_v24i10e35860_app1.docx]

The US regions are divided as follows.

Northeast: Connecticut, Maine, Massachusetts, New Hampshire, Rhode Island, Vermont, New Jersey, New York, and Pennsylvania.

Northcentral: Illinois, Indiana, Michigan, Ohio, and Wisconsin, Iowa, Kansas, Minnesota, Missouri, Nebraska, North Dakota, and South Dakota.

South: Delaware, Florida, Georgia, Maryland, North Carolina, South Carolina, Virginia, Washington D.C., and West Virginia, Alabama, Kentucky, Mississippi, Tennessee, Arkansas, Louisiana, Oklahoma, Texas.

West: Arizona, Colorado, Idaho, Montana, Nevada, New Mexico, Utah, and Wyoming, Alaska, California, Hawaii, Oregon, and Washington.
